# Evaluation of left ventricular torsion using cardiac MRI. Validation of feature tracking

**DOI:** 10.1186/1532-429X-17-S1-O47

**Published:** 2015-02-03

**Authors:** Mark P Ainslie, Anna Reid, Christopher A Miller, David Clark, Lenin Francis, Matthias Schmitt

**Affiliations:** 1 Cardiology, UHSM, Manchester, UK

## Background

### LV torsion

Left ventricular torsion may be more sensitive in detecting pathology before changes in more traditional functional parameters, and the onset of symptoms. The current gold standard for evaluating torsion is CMR, but a wide variety of techniques exist and as yet the advantages and disadvantages remain to be determined. The use of feature tracking software offers a method using standard untagged SSFP images with a potential benefit of shorter scan time.

Torsional shear angle is probably the best current measure of true torsion, but requires several parameters to be defined and no commercially available software exists to calculate this. The purpose of the study was to validate the CMR derived torsion,using an in-house developed software program using data from feature-tracking.

## Methods

15 healthy volunteers underwent a low-dose dobutamine stress CMR (baseline, 7.5mcg/kg/min, 15mcg/kg/min). In-house developed software CMRTorsion is an Excel-based macro programme using Visual Basic that facilitates the calculation and graphical representation of principal strains, twist, torsion and circumferential longitudinal shear from processed bSSFP cine images obtained by a Siemens 1.5T Avanto CMR scanner. This was further refined to allow data derived from feature tracking software to be processed.

## Results

The mean age of the volunteers was 40.3 ± 16.6 years, 7 male. The mean LV ejection fraction was 61.6 ± 3% and end diastolic volume 155.6 ± 30.2 mls. A chronotropic response was observed with a mean HR of 68±9bpm at baseline and 102±20bpm at peak stress, p<0.01.

Twist and Torsion were both calculated from data derived from CMR feature tracking (CMR-FT).

**Figure 1:**
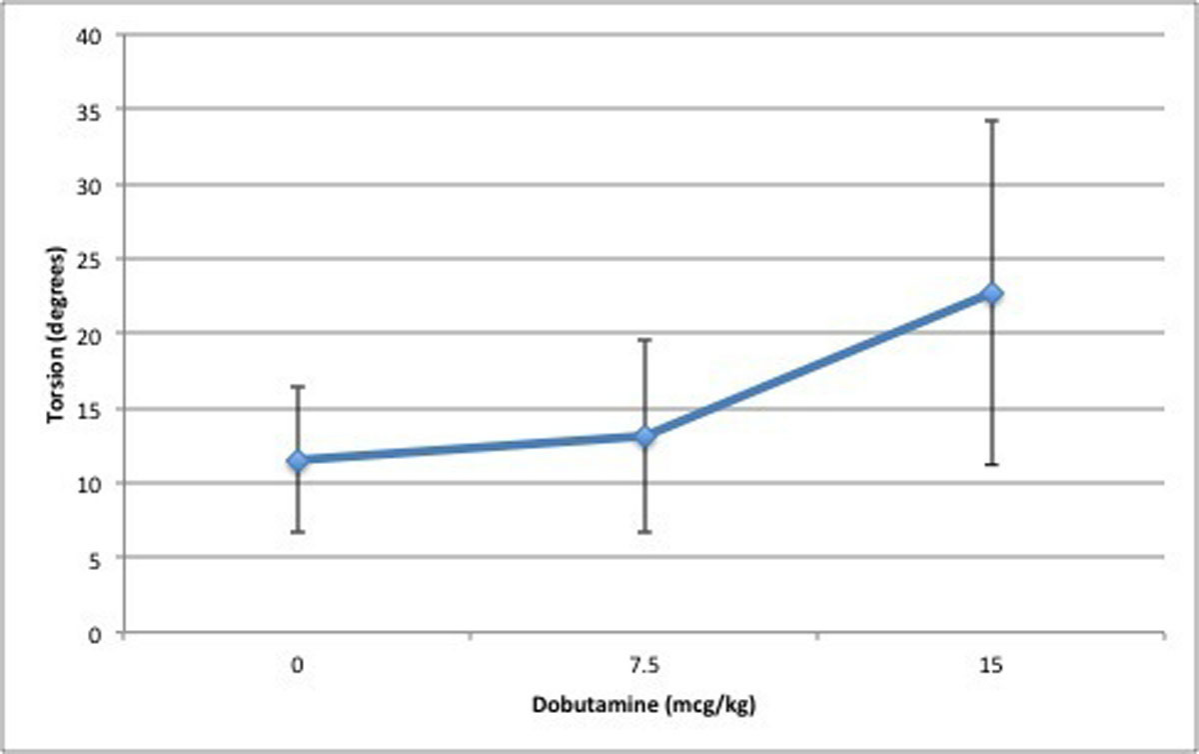
Effect of increasing dobutamine does on Torsion measured by CMR-FT

The effect of increasing dobutamine on torsion is illustrated in figure 1. A one-way repeated measures ANOVA was conducted to compare the effect of dobutamine on Twist and torsion (table 1).

**Table 1:** Effect of dobutamine on twist and torsion

	Dobutamine	Dobutamine	Dobutamine	ANOVA	Significance	Pearson correlation	Significance
	0 mcg	7.5mcg	15mcg	F	p	r	p

Twist	11.53±4.9	13.1±6.4	22.7±11.5	(1.56, 14.02) = 3.4	0.07	0.497	0.001

Torsion	5.3±2	6.7±4.2	7.7±3.5	(1.36, 10.86) = 1.08	0.35	0.288	0.002

## Conclusions

CMR-FT showed a strong positive correlation with increasing dose of dobutamine for twist and a weak positive correlation for torsion, p 0.001 and 0.075 respectively.

## Conclusions

The in-house developed software when used in conjunction with CMR-FT was able to measure twist and torsion, with the expected increase in these indices with dobutamine concentration. The in-house developed software requires further validation in disease states but may allow the identification of disease earlier than taditional parameters of LV function.

## Funding

British Heart Foundation.

